# No Increase in Colon Cancer Risk Following Induction with Neu5Gc-Bearing Rabbit Anti-T Cell IgG (ATG) in Recipients of Kidney Transplants

**DOI:** 10.3390/cancers10090324

**Published:** 2018-09-12

**Authors:** Jean-Paul Soulillou, Caner Süsal, Bernd Döhler, Gerhard Opelz

**Affiliations:** 1 Centre de Recherche en Transplantation et Immunologie UMR 1064, INSERM, Université de Nantes, 44093 Nantes, France; jean-paul.soulillou@univ-nantes.fr; 2 Institut de Transplantation Urologie Néphrologie (ITUN), CHU Nantes, 44093 Nantes, France; 3 Institute of Immunology, Heidelberg University, 69120 Heidelberg, Germany; caner.suesal@med.uni-heidelberg.de (C.S.); gerhard.opelz@med.uni-heidelberg.de (G.O.)

**Keywords:** colon cancer, anti-Neu5Gc, red meat

## Abstract

Because of a mutation of the gene allowing the synthesis of the Neu5Gc form of neuraminidic acid, humans lack the Neu5Gc present in other mammals and develop anti-Neu5Gc. However, humans can absorb dietary Neu5Gc and normal colon epithelium displays minute amounts of Neu5Gc. The potential “physiological” formation of in situ immune complexes has been proposed as a risk factor for colon cancer and as the link between red meat-rich diet and colon carcinoma. In this article, we took advantage of evidence that polyclonal rabbit IgG (ATG) elicits an immune response against Neu5Gc and we consulted a large data base of allograft recipients treated or not with animal-derived IgG to discuss this hypothesis. Based on data from 173,960 and 38,505 patients without and with ATG induction, respectively, we found no evidence that exposure to higher levels of anti-Neu5Gc is associated with a higher incidence of colon carcinoma.

Most mammalian cell surfaces display two major sialic acids, N-acetylneuraminic acid (Neu5Ac) and N-glycolylneuraminic acid (Neu5Gc) [1,2]. Due to a mutation in the cystidine monophosphate acetyl hydroxylase which occurred after evolutionary divergence from great apes, humans cannot synthetize the de-acetylated form Neu5Gc and express only the acetylated form Neu5Ac [3,4]. Neu5Gc can however be taken up through diet containing mammal milk or meat and leads to the formation of anti-Neu5Gc antibodies in the blood in the first year of life [5,6]. These anti-Neu5Gc antibodies are maintained during the whole life of a human being at a substantial level [7]. Whereas evolution has likely censured possible deleterious effects of the diet-derived anti-Neu5Gc antibody, animal-derived tissues such as engineered pig skin for severe burns [8] or molecules such as rabbit anti-thymocyte globulin (ATG), which contain Neu5Gc in mass spectrometry analysis [9], are able to trigger a vigorous memory-like rise of anti-Neu5Gc antibodies [10], in addition to antibodies directed at the IgG protein backbone or at other sugar such as α1,3Gal. This immune response differs from the diet-induced response by a shift of the anti-Neu5Gc repertoire with the appearance of antibodies recognizing new epitopes on the highly diverse antigenic sites of Neu5Gc bearing glycan [8,11,12,13]. Despite being strongly immunosuppressed by means of immunosuppressive medication, recipients of a kidney transplant [9,13] who received ATG as induction therapy immediately after surgery, exhibit several months after administration of ATG higher titers of anti-Neu5Gc antibodies than transplant recipients without ATG induction.

Because epithelial cells of normal human tissues, such as in the colon, also express detectable diet-derived Neu5Gc [12], it has been hypothesized that, beside various other possible etiologic factors [14], anti-Neu5Gc antibodies could contribute to carcinogenesis through the chronic inflammation resulting from in situ formation of immune complexes [15,16,17]. In mice, anti-Neu5Gc antibodies have ambivalent effects on tumor growth [18]. In this context, the “xenosialitis” related to anti-Neu5Gc antibodies has been proposed as a possible explanation for the association between malignancy of the colon and high red meat consumption which is the major source of dietary Neu5Gc [14]. Proof for an association of anti-Neu5Gc levels with the incidence of colon cancer would require the analysis of anti-Neu5Gc antibody levels in a huge cohort of individuals with or without colon cancer. The proposed causal role of anti-Neu5G in inducing tumors in mice [17] is speculative in humans. Although no difference was observed using ELISA, higher anti-Neu5Gc binding synthetic Neu5Gc glycans have been recently reported in a series of 71 patients with colon carcinoma [19]. These findings did not attain statistical power and the proposed causal role of Neu5Gc accumulation in tumors [5] remains speculative. Furthermore, as diet-induced anti-Neu5Gc antibodies are present in virtually all humans, and as the risk of xenosialitis also concerns activation of endothelial cells which as epithelial cells can display low level of Neu5Gc [9,20], the hypothesis implies permissive evolutionary pressure favoring the Neu5Gc negative phenotype.

Studies that are aimed at demonstrating a possible link between the incidence of colon cancer and anti-Neu5Gc might benefit from recent information on increased levels of anti-Neu5Gc elicited in patients who received animal-derived polyclonal rabbit ATG and who provide a large informative patient cohort that allows the assessment of a possible deleterious “xenosialitis” effect of anti-Neu5Gc on colonic epithelium.

It has been shown that rabbit ATG induces a vigorous anti-Neu5Gc response in humans [10,12]. In spite of receiving potent immunosuppressive treatment, compared to patients without ATG induction, patients who received rabbit ATG during the first weeks following kidney transplantation have been also shown to develop anti-Neu5Gc antibodies that circulate for months or years in the blood [9]. Similar to the patients who did not receive immunosuppressive treatment, these patients also displayed a shift in the anti-Neu5Gc antibody repertoire, showing new specificities that were not present before the administration of ATG [12,13].

We took advantage of the two key variables precisely documented in a large population of more than 200,000 kidney transplant recipients as part of the Collaborative Transplant Study (CTS) [21], namely (i) the absence or presence of post transplantation induction therapy with rabbit ATG and (ii) the absence or presence of a colon cancer, to analyze a possible link between anti-Neu5Gc exposure and colon cancer. The work of the CTS is approved by the ethics committee of the Medical Faculty of Heidelberg University (No. 083/2005).

As shown in Figure 1, the administration of rabbit ATG to 38,505 kidney recipients (76 with colon cancer) as post-transplant induction therapy, which is known to be associated with significantly increased levels of Neu5Gc compared to no ATG induction [9,10], was not associated with a higher incidence of colon cancer during 10 years of follow-up (0.543%) when compared with the incidence in 173,960 patients (446 with colon cancer) who received a kidney graft without rabbit ATG induction treatment, based on a cumulative colon cancer incidence of 0.547%.

Retrospective analyses of data regarding the study of variables possibly involved in cancer etiology [22,23] depend on the accuracy of data collection. In this respect, our observation benefits from the accuracy of recording two clinically well identified variables: ATG induction and development of colon cancer. The CTS registry has collected data on cancer in transplant recipients since the early 1980s. Because underreporting of tumors was recognized as a potential problem early on, particular emphasis has been placed on the accuracy and completeness of reporting and all centers included in this analysis have confirmed yearly that their cancer data submitted to CTS were complete and accurate. A limitation of our analysis is that the patients included were not tested for Neu5Gc antibodies. Not unusual for large population-based studies, we relied on data by inference from publications showing that ATG treatment of transplant recipients induces a significant and long lasting presence of Neu5Gc antibodies. In addition, the meat intake was not known. Although the content of Neu5Gc in the diet does not correlate with the level of anti-Neu5Gc [19], it might conceivably influence the degree of Neu5Gc deposit on epithelia.

In summary, our findings obtained in a large cohort of over 200,000 kidney transplant patients including 522 patients with colon cancer do not support the hypothesis that long term over-exposure to anti-Neu5Gc antibodies, in our analysis as a result of ATG treatment, triggers a malignancy in the colon. ATG-treated patients did not develop colon cancer at an increased rate even though their immune system was suppressed by the administration of systemic immunosuppressive drugs. Definitive assessment of an etiological role of anti-Neu5Gc in colon cancer will remain a matter of debate until statistically powered studies become available in which all interfering parameters (anti-Neu5Gc antibodies and Neu5Gc deposits) will be controlled.

## Figures and Tables

**Figure 1:**
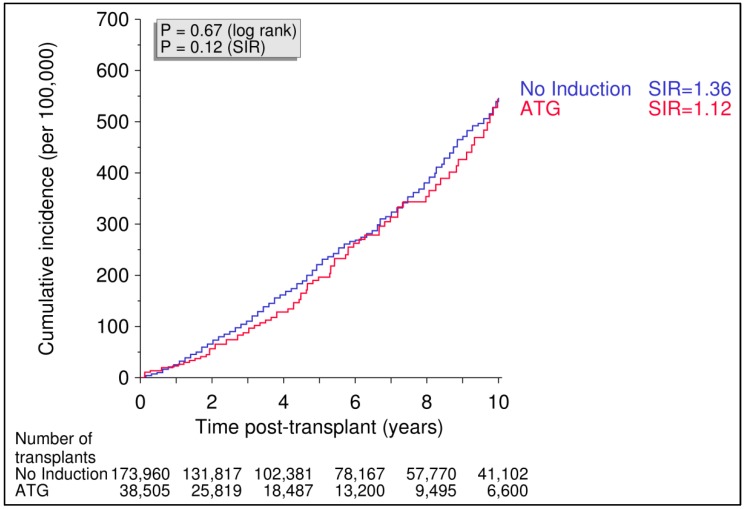
Cumulative incidence of colon cancer in recipients with or without induction therapy with polyclonal rabbit anti-T cell antibodies (ATG). Renal transplants performed from 1990 to 2016 were analyzed. Log rank *p* value for Kaplan–Meier analysis. Standardized incidence ratio (SIR) is adjusted for recipient age, gender, geographic origin, and year of tumor diagnosis.
